# Thrombopoietin: a potential diagnostic indicator of immune thrombocytopenia in pregnancy

**DOI:** 10.18632/oncotarget.7106

**Published:** 2016-01-31

**Authors:** Xu Zhang, Yajing Zhao, Xiaoqing Li, Panpan Han, Fangmiao Jing, Zhangyuan Kong, Hai Zhou, Jihua Qiu, Lizhen Li, Jun Peng, Ming Hou

**Affiliations:** ^1^ Department of Hematology, Qilu Hospital, Shandong University, Jinan, Shandong, China; ^2^ School of Medicine, Zhejiang University, Hangzhou, Zhejiang, China; ^3^ Key Laboratory of Cardiovascular Remodeling and Function Research, Chinese Ministry of Education and Chinese Ministry of Health, Qilu Hospital, Shandong University, Jinan, Shandong, China; ^4^ Shandong Provincial Key Laboratory of Immunohematology, Qilu Hospital, Shandong University, Jinan, Shandong, China

**Keywords:** immune thrombocytopenia, pregnancy, diagnosis, thrombopoietin, platelet, Pathology Section

## Abstract

To evaluate whether the serum thrombopoietin levels in pregnancy-associated immune thrombocytopenia (ITP) differ from those in gestational thrombocytopenia, and reveal the possibility of thrombopoietin serving as a marker for differential diagnosis. Serum thrombopoietin concentration was determined in ITP in pregnancy (*n* = 35), gestational thrombocytopenia (*n* = 31), healthy pregnancy (*n* = 32), age-matched nonpregnant ITP (*n* = 32) and nonpregnant healthy controls (n = 35) by ELISA. The serum thrombopoietin level of ITP in pregnancy (1283 ± 646 pg/mL) was significantly higher than gestational thrombocytopenia (187 ± 64 pg/mL) (*P* < 0.01), although the platelet counts of these two disorders may overlap. Twenty-nine of 35 patients with ITP in pregnancy had thrombopoietin values >500 pg/mL, whereas none of the gestational thrombocytopenia patients' thrombopoietin levels exceeded 500 pg/mL. In addition, ITP in pregnancy presented a markedly higher thrombopoietin level than nonpregnant ITP (88 ± 41 pg/mL) (*P* < 0.01), indicating that the pathogenesis of pregnant and nonpregnant ITP was different. Our findings suggest that measurement of serum thrombopoietin concentration provides valuable diagnostic information for differentiating ITP in pregnancy from gestational thrombocytopenia. Thrombopoietin represents a reliable marker for ITP in pregnancy.

## INTRODUCTION

Thrombopoietin (TPO), the ligand of c-Mpl protein, is the major physiological regulator of megakaryocyte maturation and platelet production.[1, 2] TPO enhances megakaryocyte colony formation; increases the size, number and ploidy of developing megakaryocytes; and results in increased platelet production.[3, 4] In turn, platelets become desialylated as they circulate and age in blood, the desialylated platelets bind to the hepatic Ashwell-Morell receptor (AMR) and induce hepatic TPO gene transcription and translation.[5] TPO is expressed mainly in liver, and cleared from the circulation blood by binding to the c-Mpl ligands on platelets, megakaryocytes and their progenitors.[6] With a largely constitutive hepatic TPO production, the circulating TPO level is controlled by the total cell mass of the megakaryocyte lineage that expresses c-Mpl receptor.[7]

Up to 10% of all pregnancies present thrombocytopenia, most of which are caused by gestational thrombocytopenia (GT).[8] GT, also known as incidental thrombocytopenia of pregnancy, is a benign thrombocytopenia appears mostly in the late stage of pregnancy, not associated with adverse outcomes to either the mother or fetus.[9] Immune thrombocytopenia (ITP) is an autoimmune hematological disease characterized by thrombocytopenia caused by accelerated platelet destruction and suppression of platelet production, [10, 11] which is common in young women.[12] ITP appears in 1 to 2 pregnancies per thousand, and it may lead to bleeding complications in both the mother and her infant.[13] However, the diagnosis of ITP in pregnancy is still indefinite so far, no laboratory test has been established to distinguish it from GT.

Previous studies have shown that TPO levels are mildly elevated or within the normal range in ITP patients.[14, 15] However, little data are available about the TPO level in pregnancy-associated ITP or in GT. We investigated serum TPO levels in pregnant women associated with ITP and GT. The first purpose of the present study was to evaluate whether or not serum TPO levels in pregnancy-associated ITP differ from those in GT, and to reveal the possibility of TPO serving as a marker for differential diagnosis. The second purpose of the study was to compare the TPO levels in pregnant *versus* nonpregnant ITP patients.

## RESULTS

The demographic and disease characteristics of patients in different groups and healthy controls were summarized in Table 1. Thirty-five nonpregnant healthy controls (22-39 years old, median age 29), 32 healthy pregnant women (21-39 years old, median age 27), 31 GT patients (18-36 years old, median age 28), 35 ITP in pregnancy patients (21-38 years old, median age 27), and 32 age-matched nonpregnant ITP patients (18-45 years old, median age 30) were enrolled in this study. The median gestational age of healthy pregnant women, GT patients and ITP in pregnancy patients were 23 weeks (range 6-41 weeks), 36 weeks (range 29-41 weeks) and 30 weeks (range 8-39 weeks), respectively. In ITP in pregnancy group, 29 patients were newly diagnosed or had persistent ITP, the other 6 patients had chronic ITP; the corresponding numbers in nonpregnant ITP group were 23 and 9. Five pregnancy-associated ITP patients received corticosteroid therapy; response (platelet count ≥30 × 10^9^/L and at least 2-fold increase of the baseline platelet count and absence of bleeding) was achieved in 4 of them. Two pregnancy-associated ITP patients received IVIg treatment, both of which had platelet counts increased to more than 50 × 10^9^/L.

**Table 1:** Demographic characteristics and TPO results of patients in different groups

-	Nonpregnant healthy control *n* = 35	Healthy pregnancy *n* = 32	GT *n* = 31	ITP in pregnancy *n* = 35	Age-matched nonpregnant ITP *n* = 32
Age (yr), median, (range)	29, (22-39)	27, (21-39)	28, (18-36)	27, (21-38)	30, (18-45)
Gestational age (wk), median, (range)	NA	23, (6-41)	36, (29-41)	30, (8-39)	NA
Platelet count (×109/L) median, (range)	256, (182-297)	232, (190-277)	81, (53-99)	18, (4-43)	21, (1-58)
TPO level (pg/mL) mean ± SD	37 ± 15	65 ± 22	187 ± 64	1283 ± 646	88 ± 41
Newly diagnosed or persistent ITP, n (%)	NA	NA	NA	29 (82.9%)	23 (71.9%)
Chronic ITP, n (%)	NA	NA	NA	6 (17.1%)	9 (28.1%)
Current ITP therapy, n (%)					
Any	NA	NA	NA	6 (17.1%)	8 (25.0 %)
Corticosteroid	NA	NA	NA	5 (14.3%)	6 (18.8%)
IVIg	NA	NA	NA	2 (5.7%)	3 (9.4%)
Rituximab	NA	NA	NA	0 (0.0%)	1 (3.1%)
Other	NA	NA	NA	0 (0.0%)	2 (6.3%)
No therapy	NA	NA	NA	29 (82.9%)	24 (75.0%)
Platelet count after delivery (×109/L), n	NA	NA	22	20	NA
median, (range)	NA	NA	241, (170-274)	74, (37-167)	NA
TPO level after delivery (pg/mL), n	NA	NA	0	8	NA
mean ± SD	NA	NA	NA	95 ± 32	NA

### TPO levels and platelet counts

**Figure 1:**
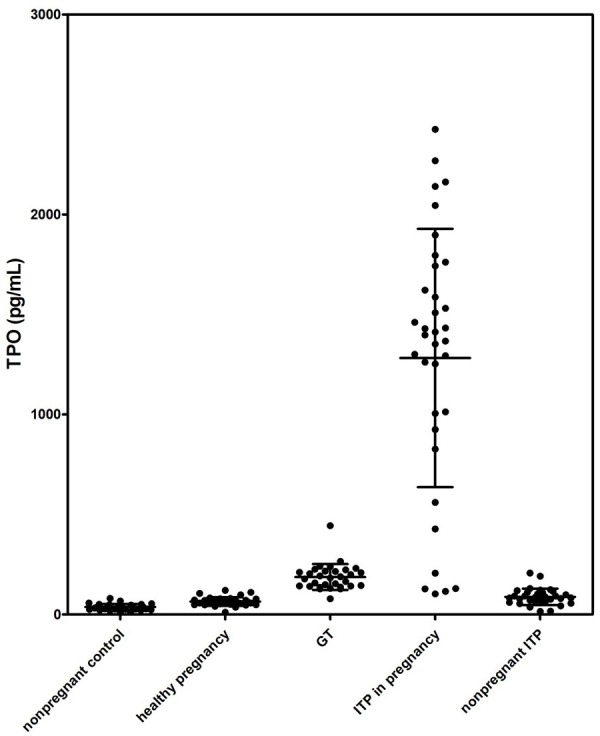
Serum TPO levels in different groups

The platelet counts and TPO levels for the different patient groups and for the healthy controls were summarized in Table 1 and Figure 1.

Serum TPO levels were 37 ± 15 pg/mL in nonpregnant healthy controls with normal platelet counts (182 to 297 × 10^9^/L, median 256 × 10^9^/L). The healthy pregnant women presented slightly decreased circulating platelet numbers (190 to 277 × 10^9^/L, median 232 × 10^9^/L) and increased TPO levels (65 ± 22 pg/mL) compared to the nonpregnant healthy controls. We further analyzed platelet counts and TPO levels in different stages of pregnancy. The platelet count showed a slight decrease in the third trimester compared to the first two, whereas the TPO level showed no significant difference (data not shown). Therefore, we can directly compare the TPO levels in different groups despite the different gestational ages. In GT patients, platelet counts (53 to 99 × 10^9^/L, median 81 × 10^9^/L) were decreased and the TPO levels (187 ± 64 pg/mL) were elevated compared to the healthy pregnancies.

With a marked lower platelet level, nonpregnant ITP displayed a TPO level (88 ± 41 pg/mL) mildly higher than nonpregnant healthy controls. In all of the 32 nonpregnant ITP patients, 23 patients presented elevated TPO levels. For the other 9 patients, TPO levels were within the normal range (mean ± 2SD of the healthy control group).

There was no significant difference in circulating platelet counts between pregnant and nonpregnant ITP patients (*P* > 0.05); however, TPO levels in pregnant ITP patients (1283 ± 646 pg/mL) were significantly higher than those in nonpregnant patients (*P* < 0.01).

**Figure 2:**
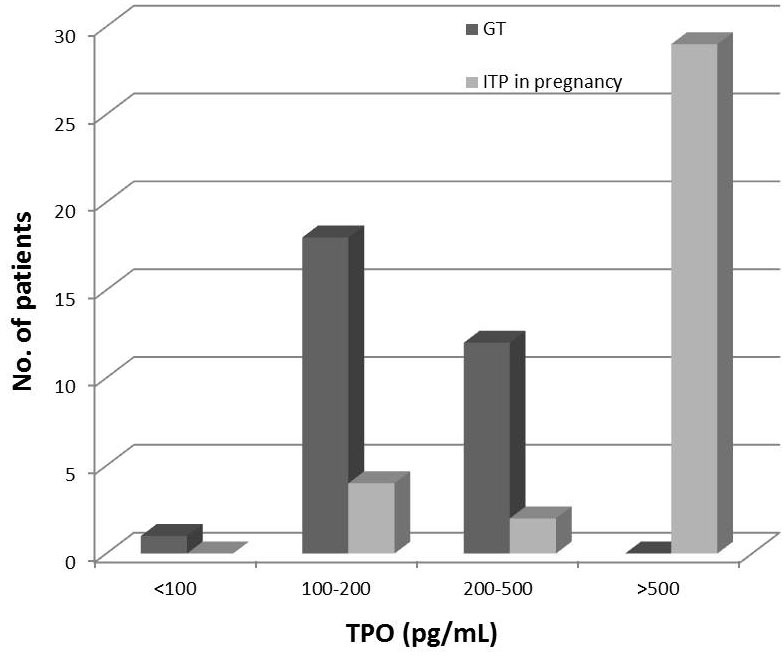
TPO levels in gestational thrombocytopenia (GT) and ITP in pregnancy patients presented in categories Twenty-nine of 35 ITP in pregnancy patients had TPO values > 500 pg/mL, while none of the GT patients' TPO level exceeded 500 pg/mL.

Compared to GT, ITP in pregnancy displayed a slightly lower platelet count (4 to 43 × 10^9^/L, median 18 × 10^9^/L), while the TPO level was significantly higher (*P* < 0.01). Twenty-nine of 35 patients with ITP in pregnancy had TPO values > 500 pg/mL, but none of the GT patients' TPO level exceeded 500 pg/mL (shown in Figure 2).

The platelet counts and TPO levels after delivery were also obtained in some of the pregnancy-associated ITP and GT patients (shown in Table 1). Platelet counts of all the GT patients resolved to normal after delivery. In contrast, most of the pregnancy-associated ITP patients still had lower platelet counts after delivery. In pregnancy-associated ITP group, TPO levels after delivery (95 ± 32 pg/mL) were significantly lower than during pregnancy.

### Bone marrow megakaryocyte numbers

The bone marrow data of 27 nonpregnant ITP patients and 23 ITP in pregnancy patients were collected in our study. The results were summarized in Table 2. Most of the nonpregnant ITP patients (17/27) had increased numbers of megakaryocytes, while the rest (10/27) had normal numbers of megakaryocytes. Conversely, among 23 patients with ITP in pregnancy, 14 had decreased numbers of megakaryocytes, 5 had normal numbers and 4 had increased numbers of megakaryocytes. Among the 14 patients with decreased megakaryocyte numbers, none of them had abnormality in granulopoiesis, 12 patients had normal and 2 had active erythropoiesis. Meanwhile, megakaryocytic abnormalities including hypolobated forms and diminished granularity were common in patients with ITP in pregnancy.

**Table 2:** Data on ITP patients included in this study

-	ITP in pregnancy *n* = 35	Nonpregnant ITP *n* = 32
Platelet count (×109/L), median (range)	18 (4-43)	21 (1-58)
TPO level (pg/mL), mean ± SD	1283 ± 646	88 ±41
Platelet autoantibodies, available number	*n* = 25	*n* = 32
Anti-GP I bIX positive, n (%)	1 (4.0%)	5 (15.6%)
Anti-GP II bIIIa positive, n (%)	5 (20.0%)	8 (25.0%)
Double positive, n (%)	5 (20.0%)	7 (21.9%)
Negative, n (%)	14 (56.0%)	12 (37.5%)
Bone marrow megakaryocytes, available number	*n* = 23	*n* = 27
Decreased, n (%)	14, (60.9%)	0, (0%)
Normal, n (%)	5, (21.7%)	10, (37.0%)
Increased, n (%)	4, (17.4%)	17, (63.0%)

### Platelet autoantibodies

Platelet glycoprotein-specific autoantibodies were detected in 25 ITP in pregnancy patients and 32 nonpregnant ITP patients (shown in Table 2). A lower percent of ITP in pregnancy patients (44.0%) was positive compared to the nonpregnant ITP group (62.5%), but the difference was not significant. There was no significant difference in the frequency of platelet glycoprotein-specific autoantibodies between these two groups, and we did not find a correlation between the presence or absence of detectable platelet autoantibodies and TPO levels in either pregnant or nonpregnant ITP group.

## DISCUSSION

Our study demonstrated that TPO levels of ITP in pregnancy patients are extremely elevated and significantly higher than those of GT patients. Thrombocytopenia is relatively common in pregnancy. The main causes of thrombocytopenia in pregnancy can be divided into gestational thrombocytopenia (75%), secondary to hypertension disorders (15-20%), and immunological disorders of pregnancy (3-4%).[16-18] Disorders like preeclampsia, HELLP syndrome, acute fatty liver, disseminated intravascular coagulation, thrombotic thrombocytopenic purpura, and hemolytic uremic syndrome can be diagnosed based on abnormal clinical and laboratory findings. Furthermore, thrombocytopenia may also be the primary manifestation of viral infections or adverse reaction from many drugs and supplements, which should be carefully differentiated by history taking and laboratory examinations.[19] However, as for ITP first presenting in pregnancy and GT, they have often been inadvertently confused in clinical practice.[20] In the case of otherwise asymptomatic thrombocytopenia first presenting during pregnancy, bone marrow aspiration is not routinely performed. Hematologists and obstetricians differentiate ITP from GT mainly based on onset time and platelet count.[20] Significant thrombocytopenia in the first or early-second trimester of pregnancy, with a declining platelet count as gestation progresses, is most consistent with the diagnosis of ITP. In contrast, mild thrombocytopenia developing late in the third trimester and not associated with hypertension or proteinuria is usually diagnosed as GT, in which cases the platelet count rarely drops below 70,000/μL.[9] However, early onset thrombocytopenia and nadir platelet counts of less than 70,000/μL can also be seen in more severe forms of GT, and mild types of ITP may present platelet counts higher than 70,000/μL, in which case these two disorders become indistinguishable. As the most common cause of thrombocytopenia in pregnancy, GT appears to have no adverse effect on either the mother or the fetus, and it resolves spontaneously within 1 to 2 months after delivery.[21] Conversely, ITP in pregnancy may lead to bleeding complications in both mothers and their infants.[12, 13] Besides the possible hemorrhage of ITP patients during delivery or post-partum, the neonates of mothers with ITP may also develop thrombocytopenia, as a result of the trans placental passage of maternal antiplatelet IgG.[22] It is necessary to observe the conditions of ITP patients closely and provide proper care and treatment. Therefore, accurate differentiation of ITP in pregnancy from GT is of crucial importance in clinical practice. We showed in the study that the TPO levels of ITP in pregnancy patients were significantly higher compared to those of GT patients. The significance becomes obvious when patients are ranked according to TPO level. (Figure 2) In our study, 29/35 ITP in pregnancy patients had TPO values > 500 pg/mL, but none of the GT patients' TPO level exceeded 500 pg/mL. These results indicate that measurement of the TPO level may give additional information in differentiating ITP from GT. Patients with TPO levels of 500 pg/mL or over need to be examined and observed carefully, since the thrombocytopenia is more likely to be caused by ITP.

TPO levels in pregnant and nonpregnant ITP patients were significantly different despite equivalent platelet counts. Numerous studies over the past 2 decades have documented that TPO levels are high when thrombocytopenia is due to megakaryocyte deficiency and low when it is due to increased platelet destruction.[15, 23, 24] The serum TPO levels are regulated by both circulating platelets and bone marrow megakaryocytes. In thrombocytopenic patients with bone marrow hypoplasia, circulating TPO levels are characteristically high due to low amount of total c-Mpl expressing cellular mass and therefore defective uptake of TPO. In contrast, despite similar degrees of thrombocytopenia, nonpregnant ITP patients have increased or normal bone marrow megakaryocyte numbers and the circulating TPO levels are relatively low. Taking the previously published studies and our data together, it could be inferred that the pathogenesis of ITP in pregnancy might be different from that of nonpregnant ITP. We hypothesized that there is megakaryocyte hypoplasia in patients of ITP in pregnancy. Based on this conjecture, we further performed bone marrow examinations of pregnant and nonpregnant ITP patients. Most of the nonpregnant ITP patients (17/27) had increased numbers of megakaryocytes, while the rest had normal numbers of megakaryocytes. Conversely, among 23 patients with ITP in pregnancy, 14 had decreased numbers of megakaryocytes, 5 had normal numbers and 4 had increased numbers of megakaryocytes. These data showed that more than half of the ITP patients in pregnancy underwent megakaryocyte hypoplasia. This finding needs to be replicated with a larger study. In addition, platelet glycoprotein-specific autoantibodies were determined in both pregnant and nonpregnant ITP groups. There was no significant difference in the frequency of platelet glycoprotein-specific autoantibodies between these two groups. We further analyzed the TPO levels in newly diagnosed ITP during pregnancy and chronic ITP diagnosed before pregnancy. In a total of 6 chronic ITP patients, TPO levels were lower than 500 pg/mL in 4 and higher than 500 pg/mL in 2 of them. In the 29 ITP patients first diagnosed during pregnancy, only 1 patient displayed TPO level of lower than 500 pg/mL, while the others had TPO levels significantly higher. After delivery, platelet counts slightly increased in most of the ITP patients, meanwhile, the TPO levels significantly decreased, indicating that the pathogenesis during and after pregnancy were different. Unfortunately, since only a limited number of patients had chronic ITP, it could not be determined whether there is a difference in the level of TPO in patients with newly diagnosed, persistent and chronic ITP in this study. Further research will be needed to make subgroup analysis in the future. Meanwhile, measuring of TPO levels in chronic ITP patients before, during and after pregnancy would be meaningful to further clarify the role of pregnancy in the course of ITP.

The healthy pregnant women presented slightly decreased circulating platelet numbers and increased TPO levels compared to the nonpregnant healthy controls, which is consistent with former studies.[25, 26] Platelet count decreases by an average of approximately 10 percent in uncomplicated pregnancies. The mechanisms of the decrease in platelet count are thought to be dilutional effects and accelerated destruction of platelets passing over the often scarred and damaged trophoblast surface of the placenta.[25] The elevation of the circulating TPO levels might be the physiological response to decreased circulating platelets, because the consumption of TPO by platelets decreases.

Gestational thrombocytopenia is characterized by decreased platelet count and increased platelet volume compared to healthy pregnancy. The precise etiology of GT remains unknown, but it has been suggested to be a result of various mechanisms, including hemodilution during pregnancy and increased activation and clearance of platelets.[8, 17] We found that serum TPO levels were elevated in GT compared to healthy pregnancies. This elevation is most likely to be compensatory due to the reduced platelet count.

Consistent with previous reports,[14, 15] the present study revealed mildly elevated TPO levels of patients with nonpregnant ITP compared to nonpregnant healthy controls. Although the circulating platelet counts of nonpregnant ITP patients were markedly lower than the healthy controls, the TPO levels were not as high as would be predicted by their platelet counts. Former reports have suggested that the relatively low TPO levels in ITP might be the result of preserved megakaryocyte mass in the bone marrow of patients with accelerated platelet destruction.[24, 27] With an increased number of c-Mpl positive megakaryocytes and continuous production of new platelets, the TPO uptake in nonpregnant ITP patients would not extremely decrease. In addition, Chang M et al[28] suggested that the platelets of ITP have a higher capacity to take up and degrade TPO.

There are some limitations in our study, including limited sample and sampling bias of single-centre study. Larger prospective multicenter studies are needed to further confirm our findings.

In conclusion, we demonstrated that ITP in pregnancy displayed a significantly higher TPO level compared to GT. Although the exact mechanism for elevated TPO levels of ITP in pregnancy patients remains unclear, measurement of its concentration could be an important diagnostic tool for differentiate ITP in pregnancy from GT. In addition, the pathogenesis of ITP in pregnancy might be different from that of nonpregnant ITP, the exact mechanism will need to be elucidated with further research.

## MATERIALS AND METHODS

### Patients

Blood samples were obtained from ITP in pregnancy patients, GT patients, healthy pregnant women, age-matched nonpregnant ITP patients, and nonpregnant healthy controls at Qilu Hospital, Shandong University. Inclusion criteria were: 1) female, 15 years to 49 years old; 2) no underlying disease before pregnancy or comorbidity during pregnancy for the pregnant individuals; 3) ITP or GT diagnosed according to the diagnostic criteria described previously.[9, 29] Nonpregnant ITP was diagnosed clinically as thrombocytopenia with normal or increased bone marrow megakaryocytes and no concurrent abnormality that could otherwise explain thrombocytopenia. Mild thrombocytopenia that first became apparent in the second to third trimester of pregnancy with exclusion of other causes of thrombocytopenia during pregnancy was diagnosed as GT. A diagnosis of ITP in pregnancy was made when the familial and personal history, physical examination and laboratory examinations did not suggest other etiologies for the thrombocytopenia, with exclusion of other autoimmune/immunodeficiency disorders, inherited thrombocytopenia, liver disease, drugs, aplastic anemia, myelodysplastic syndrome, malignancies, gestational thrombocytopenia, preeclampsia, HELLP syndrome, DIC, folate deficiency and antiphospholipid antibody syndrome. ITP patients (both pregnant and nonpregnant) who had been treated with recombinant human TPO or TPO-receptor agonist within 3 months were excluded. The ITP treatment information of pregnant and nonpregnant ITP patients within 30 days before sample collection was obtained. This study was approved by the Medical Ethical Committee of Qilu Hospital, Shandong University. Informed consent was obtained from each patient.

Ethylenediaminetetraacetic acid (EDTA)-anticoagulated whole blood and serum samples were collected during periods of thrombocytopenia. Platelet count was determined by complete blood count (CBC).

### TPO assay

After clotting and centrifugation, serum samples were stored at −40°C until use. Thrombopoietin concentration was determined by using a commercially available ELISA kit (Quantikine Human TPO Immunoassay; R&D Systems, Minneapolis, USA) as previously described.[15] Briefly, 200 μL of recombinant human TPO standard, serum samples or blank were added in duplicates to the wells of a microtiter plate precoated with an anti-TPO monoclonal antibody. The plate was incubated for 3h at room temperature. After washing, 200μL of horseradish peroxidase conjugated anti-TPO antibody was added and incubated for 1h at room temperature. The color was developed by using tetramethylbenzidine as substrate. The reaction was stopped by adding 50 μL of acid solution to each well, and the absorbance was recorded at 450/650 nm wavelengths. The sample TPO concentration was calculated from the corresponding standard curve.

### Bone marrow morphology

Bone marrow was aspirated from the posterior superior iliac spine and stained according to Wright-Giemsa. Bone marrow megakaryocyte numbers were determined in double blind. The number of megakaryocytes was rated as normal (1 megakaryocyte per 1 to 3 low power fields), increased ( > 2 megakaryocytes per low power field), or decreased (1 megakaryocyte per 5 to 10 low power fields).[30] Megakaryocyte morphology was studied with a 100 × objective.

### Platelet autoantibody assay

Platelet glycoprotein-specific autoantibodies were determined by modified monoclonal antibody specific immobilization of platelet antigens (MAIPA) assay as previously described.[31] An absorbance higher than the mean absorbance + 3 SD recorded for the controls was considered as positive.

### Statistical analysis

The mean value ± SD is reported for continuous variables in the text, categorical variables are expressed as count and percentage. The probability of differences between unpaired variables being statistically significant was determined by ANOVA followed by Tamhane's T2 post hoc test. *P* value < 0.05 was taken as the level of significance.

## References

[1] Lok S, Kaushansky K, Holly RD, Kuijper JL, Lofton-Day CE, Oort PJ, Grant FJ, Heipel MD, Burkhead SK, Kramer JM, Bell LA, Sprecher CA, Blumberg H, Johnson R, Prunkard D, Ching AFT (1994). Cloning and expression of murine thrombopoietin cDNA and stimulation of platelet production *in vivo*. Nature.

[2] de Sauvage FJ, Hass PE, Spencer SD, Malloy BE, Gurney AL, Spencer SA, Darbonne WC, Henzel WJ, Wong SC, Kuang WJ, Oles KJ, Hultgren B, Solberg LA, Goeddel DV, Eaton DL (1994). Stimulation of megakaryocytopoiesis and thrombopoiesis by the c-Mpl ligand. Nature.

[3] Bartley TD, Bogenberger J, Hunt P, Li YS, Lu HS, Martin F, Chang MS, Samal B, Nichol JL, Swift S, Johnson MJ, Hsu RY, Parker VP, Suggs S, Skrine JD, Merewether LA (1994). Identification and cloning of a megakaryocyte growth and development factor that is a ligand for the cytokine receptor Mpl. Cell.

[4] Kaushansky K, Lok S, Holly RD, Broudy VC, Lin N, Bailey MC, Forstrom JW, Buddle MM, Oort PJ, Hagen FS, Roth GJ, Papayannopoulou T, Foster DC (1994). Promotion of megakaryocyte progenitor expansion and differentiation by the c-Mpl ligand thrombopoietin. Nature.

[5] Grozovsky R, Begonja AJ, Liu K, Visner G, Hartwig JH, Falet H, Hoffmeister KM (2015). The Ashwell-Morell receptor regulates hepatic thrombopoietin production *via* JAK2-STAT3 signaling. Nature medicine.

[6] Li J, Xia Y, Kuter DJ (1999). Interaction of thrombopoietin with the platelet c-mpl receptor in plasma: binding, internalization, stability and pharmacokinetics. British journal of haematology.

[7] Chang M, Qian JX, Lee SM, Joubran J, Fernandez G, Nichols J, Knoppel A, Buzby JS (1999). Tissue uptake of circulating thrombopoietin is increased in immune-mediated compared with irradiated thrombocytopenic mice. Blood.

[8] Burrows RF, Kelton JG (1988). Incidentally detected thrombocytopenia in healthy mothers and their infants. The New England journal of medicine.

[9] McCrae KR (2010). Thrombocytopenia in pregnancy. Hematology Am Soc Hematol Educ Program.

[10] Cines DB, Bussel JB, Liebman HA, Luning Prak ET (2009). The ITP syndrome: pathogenic and clinical diversity. Blood.

[11] Toltl LJ, Arnold DM (2011). Pathophysiology and management of chronic immune thrombocytopenia: focusing on what matters. British journal of haematology.

[12] Kelton JG (2002). Idiopathic thrombocytopenic purpura complicating pregnancy. Blood Rev.

[13] Stavrou E, McCrae KR (2009). Immune thrombocytopenia in pregnancy. Hematol Oncol Clin North Am.

[14] Kosugi S, Kurata Y, Tomiyama Y, Tahara T, Kato T, Tadokoro S, Shiraga M, Honda S, Kanakura Y, Matsuzawa Y (1996). Circulating thrombopoietin level in chronic immune thrombocytopenic purpura. Br J Haematol.

[15] Hou M, Andersson PO, Stockelberg D, Mellqvist UH, Ridell B, Wadenvik H (1998). Plasma thrombopoietin levels in thrombocytopenic states: implication for a regulatory role of bone marrow megakaryocytes. Br J Haematol.

[16] Myers B (2012). Diagnosis and management of maternal thrombocytopenia in pregnancy. Br J Haematol.

[17] Shehata N, Burrows R, Kelton JG (1999). Gestational thrombocytopenia. Clin Obstet Gynecol.

[18] Burrows RF, Kelton JG (1990). Thrombocytopenia at delivery: a prospective survey of 6715 deliveries. American journal of obstetrics and gynecology.

[19] Gernsheimer TB (2012). Thrombocytopenia in pregnancy: is this immune thrombocytopenia or…? Hematology / the Education Program of the American Society of Hematology American Society of Hematology Education Program.

[20] McCrae KR, Bussel JB, Mannucci PM, Remuzzi G, Cines DB (2001). Platelets: an update on diagnosis and management of thrombocytopenic disorders. Hematology Am Soc Hematol Educ Program.

[21] Crowther MA, Burrows RF, Ginsberg J, Kelton JG (1996). Thrombocytopenia in pregnancy: diagnosis, pathogenesis and management. Blood Rev.

[22] Gill KK, Kelton JG (2000). Management of idiopathic thrombocytopenic purpura in pregnancy. Seminars in hematology.

[23] Chang M, Suen Y, Meng G, Buzby JS, Bussel J, Shen V, van de Ven C, Cairo MS (1996). Differential mechanisms in the regulation of endogenous levels of thrombopoietin and interleukin-11 during thrombocytopenia: insight into the regulation of platelet production. Blood.

[24] Emmons RV, Reid DM, Cohen RL, Meng G, Young NS, Dunbar CE, Shulman NR (1996). Human thrombopoietin levels are high when thrombocytopenia is due to megakaryocyte deficiency and low when due to increased platelet destruction. Blood.

[25] Fay RA, Hughes AO, Farron NT (1983). Platelets in pregnancy: hyperdestruction in pregnancy. Obstet Gynecol.

[26] Frolich MA, Datta S, Corn SB (1998). Thrombopoietin in normal pregnancy and preeclampsia. Am J Obstet Gynecol.

[27] Nichol JL (1998). Endogenous TPO (eTPO) levels in health and disease: possible clues for therapeutic intervention. Stem Cells.

[28] Chang M (1997). Higher uptake and degradation of thrombopoietin (TPO) by MPL megakaryocytes (MK) and platelets (PLT) in immune thrombocytopenic compared to irradiated thrombocytopenic mice: Significant correlation to circulating TPO levels. Blood.

[29] Provan D, Stasi R, Newland AC, Blanchette VS, Bolton-Maggs P, Bussel JB, Chong BH, Cines DB, Gernsheimer TB, Godeau B, Grainger J, Greer I, Hunt BJ, Imbach PA, Lyons G, McMillan R (2010). International consensus report on the investigation and management of primary immune thrombocytopenia. Blood.

[30] Louwes H, Zeinali Lathori OA, Vellenga E, de Wolf JT (1999). Platelet kinetic studies in patients with idiopathic thrombocytopenic purpura. The American journal of medicine.

[31] Hou M, Peng J, Shi Y, Zhang C, Qin P, Zhao C, Ji X, Wang X, Zhang M (2003). Mycophenolate mofetil (MMF) for the treatment of steroid-resistant idiopathic thrombocytopenic purpura. European journal of haematology.

